# lncRNA DLEU2 promotes gastric cancer progression through ETS2 via targeting miR-30a-5p

**DOI:** 10.1186/s12935-021-02074-9

**Published:** 2021-07-14

**Authors:** Shuyi Han, Yan Qi, Yihui Xu, Min Wang, Jun Wang, Jing Wang, Mingjie Yuan, Yanfei Jia, Xiaoli Ma, Yunshan Wang, Xiangdong Liu

**Affiliations:** 1 Jinan Central Hospital Affiliated to Shandong First Medical University, 115 Jie Fang Road, Jinan, 250013 Shandong People’s Republic of China; 2 Department of Clinical Laboratory, Qingdao Municipal Hospital, Qingdao, Shandong People’s Republic of China; 3 Binzhou Medical University, Binzhou, Shandong People’s Republic of China; 4 Department of Clinical Laboratory, Shandong Provincial Hospital Affiliated To Shandong University, Jinan, Shandong People’s Republic of China; 5 Jinan Central Hospital Affiliated to Shandong University, 115 Jie Fang Road, Jinan, 250013 Shandong P.R. China

## Abstract

**Background:**

Gastric cancer (GC) remains an important cancer worldwide. Further understanding of the molecular mechanisms of gastric carcinogenesis will enhance the diagnosis and treatment of GC.

**Methods:**

The expression of DLEU2 and ETS2 was analyzed in several GC cell lines using GEPIA online analyze, qRT-PCR and immunohistochemistry. The biological behavior of GC cells was detected by CCK8, clone formation, transwell, wound healing, western blot, and flow cytometry assay. More in-depth mechanisms were studied.

**Results:**

DLEU2 was significantly up-regulated in GC tissues and cell lines. The expression of DLEU2 was significantly associated with pathological grading and TNM stage of GC patients. Furthermore, knockdown of DLEU2 inhibited the proliferation, migration, and invasion of AGS and MKN-45 cells, while overexpression of DLEU2 promoted the proliferation, migration, and invasion of HGC-27 cells. MiR-30a-5p could directly bind to the 3’ UTR region of ETS2. Moreover, DLEU2 bound to miR-30a-5p through the same binding site, which facilitated the expression of ETS2. Knockdown of DLEU2 reduced the protein level of intracellular ETS2 and inhibited AKT phosphorylation, while overexpression of DLEU2 induced the expression of ETS2 and the phosphorylation of AKT. ETS2 was highly expressed in GC tissues. The expression of ETS2 was significantly associated with age, pathological grading, and TNM stage. ETS2 overexpression promoted cell proliferation and migration of AGS and MKN-45 cells. Furthermore, ETS2 overexpression rescued cell proliferation and migration inhibition induced by DLEU2 down-regulation and miR-30a-5p up-regulation in AGS and MKN-45 cells.

**Conclusions:**

DLEU2 is a potential molecular target for GC treatment.

## Background

Gastric cancer (GC) remains an important cancer worldwide. The 2018 global cancer data released by the American Cancer Society shows that there are more than 1,000,000 new cases and an estimated 783,000 deaths (equivalent to 1 in 12 deaths globally) [1]. GC has become the fifth most common cancer and the third leading cause of cancer death [1, 2]. In addition, it is the most commonly diagnosed cancer among men [1–3]. Therefore, further revealing the potential molecular mechanisms of gastric carcinogenesis will help the diagnosis and treatment of GC [3].

Human whole-genome sequencing results show that about 1.2% of the mammalian genome is used to encode proteins [4], and most of the genome corresponds to transcriptional regulatory elements and non-coding RNAs (ncRNAs) [5]. According to size, sequence, and function, ncRNAs are divided into various subcategories, the two most famous of which are long-noncoding RNAs (lncRNAs) and microRNAs (miRNAs). LncRNAs are a type of transcript with a length of more than 200 nucleotides that cannot encode proteins [6]. LncRNAs can interact with proteins, DNA, and RNA through epigenetic modification, transcription, and post-transcriptional regulation, thereby playing an indispensable role in cells [7]. A large number of studies have discovered that lncRNAs that are dysfunctional play a role in the occurrence and progression of cancer [8–10]. LncRNAs can play the role of competitive endogenous RNAs (ceRNAs), which regulate the expression of target genes through competitive binding with miRNAs and are closely related to tumor progression [11, 12]. LncRNAs are expected to be biomarkers for the diagnosis and prognosis of GC, but the underlying mechanism remains to be clarified [13].

The host gene of lncRNA DLEU2 is located on chromosome 13, 4,995,852–50,125,541, which is a common deletion mutation in leukemia and solid tumors [14]. *DLEU2* gene encodes an lncRNA (1.0–1.8 kb)—DLEU2, which is polyadenylated and cleaved [15]. Its sequence has no homology to any other non-coding RNA. DLEU2 can act as a ceRNA to accelerate human acute myeloid leukemia by regulating miR-496/PRKACB expression [16]. Furthermore, our previous study found that DLEU2 is highly expressed in GC tissues infected with *H. pylori* [17].

Unlike lncRNAs, microRNAs (miRNAs) are generally 18–25 nucleotides in length. MiR-30a-5p is considered to be involved in the development and drug resistance of GC and is closely related to the recurrence-free and overall survival rate of GC patients [18, 19]. ETS2 is a representative member of the transcription factor ETS family, which has a DNA-binding domain at the C-terminus required for the recognition of the consensus core sequence GGAA/T [20]. ETS2 is over-expressed in breast, prostate, and renal cell carcinomas, and its deletion inhibits the survival and metastasis of these cells [16, 21, 22]. However, the roles of ETS2 in GC are not known. In this study, we aimed to investigate the expression and functional correlation of DLEU2, miR-30a-5p, and EST2 in GC.

## Methods

### Tissue collection

75 pairs of GC tissues and matched adjacent normal tissues were collected from patients who were diagnosed and underwent surgical resection at the Jinan Central Hospital. None of the patients had other serious diseases except for GC and did not receive chemotherapy or radiotherapy before operation. All collected samples were immediately snap-frozen in liquid nitrogen and stored until needed. The Histological grade was staged according to the seventh TNM staging of the International Union against Cancer/American Joint Committee on Cancer system. All research complied with the principles of the Declaration of Helsinki and was approved by the Medical Ethics Committee of the Jinan Central Hospital. The enrolled patients gave written informed consent for publication.

### Cell culture and transfections

Human GC cell lines (MKN-45, SGC-7901, HGC-27, NCI-N87, AGS, BGC823, and MGC-803) and normal gastric mucosal epithelial cell line (GES-1) were purchased from the American Type Culture Collection (ATCC, Rockville, MD) and cultured in Dulbecco’s modified Eagle medium (DMEM; 4.5 g/L D-glucose) supplemented with 10% FBS (Invitrogen, Grand Island, NY) and 1% antibiotic/antimycotic in a humidified incubator at 37 °C containing 5% CO_2_.

siRNAs targeting lncRNA DLEU2 (si-DLEU2) were designed and synthesized (RiboBio, Guangzhou, China). The sequence of si-DLEU2 was used as follows: 5'-CUCAUUGAAUACUAUCAAAAAGGAA-3'. miR-30a-5p mimics were purchased from RiboBio (Guangzhou, China). The cDNA of DLEU2 and ETS2 was synthesized by GENEWIZ and cloned into the pcDNA3.1 expression vector, respectively (GenePharma, Shanghai, China). si-DLEU2, miR-30a-5p mimics, and pcDNA3.1 vectors were transfected using Lipofectamine 2000 reagent (Invitrogen, Carlsbad, CA), according to the manufacturer’s instructions.

### Isolation of RNA and real-time PCR

Total RNA was extracted from tissue samples or cells with TRIzol reagent (Invitrogen, Carlsbad, CA) and purified according to the manufacturer’s instructions. RNA concentration was measured spectrophotometrically at an optical density of 260 nm. The Reverse Transcription Kit (Takara, Dalian, China) was used to generate cDNA. Real-time PCR was performed using SYBR® Green (Takara, Dalian, China). PCR primers were used as follows: DLEU2-Forward, 5'-GGCGGCGGGTACTTATCTC-3'; DLEU2-Reverse, 5'- CCAGGGAAGGATGTAGCTGTG-3'; GAPDH-Forward, 5'- GGTGTGAACCATGAGAAGTATGA-3'; GAPDH-Reverse, 5'- GAGTCCTTCCACGATACCAAAG-3'. Relative fold expression was calculated by 2^−ΔΔCt^ using GAPDH as an endogenous control.

### CCK8 cell viability assays

2000 cells were cultured in a 96-well plate for 0, 24, 48, and 72 h. Then, the medium was replaced with 100 μl fresh DMEM containing 10 μl CCK8 solution (Dojindo Molecular Technologies, Japan). Incubate at 37 °C for 2 h. Absorbance at 450 nm was detected by a microplate reader (BioTek Instruments, USA).

### Colony formation assays

2000 cells were plated into 6-well plates and cultured for 10 days. Then, cells were fixed with methanol and stained with 0.1% crystal violet solution. Colonies were counted and images were obtained. Colonies with at least 50 cells were considered significant.

### Transwell migration assays

1 × 10^5^ cells in 200 μl of FBS-free DMEM were seeded into the upper chamber of transwells precoated with Matrigel (BD Bioscience), and 600 μl of DMEM containing 10% FBS was added to the lower chamber. After 24 h of incubation, non-migrating or non-invasive cells remaining on the upper surface were removed with a cotton swab. Then the membranes were fixed with 4% paraformaldehyde for 30 min and stained with 0.1% crystal violet for 15 min. Three random fields were counted in each chamber using an inverted microscope (Olympus).

### Wound healing assays

A sterile plastic was used to create a wound in a single cell layer. After washing with PBS, the cells were cultured in FBS-free medium for 24 h. Five random fields of each wound were measured for quantification and images were obtained.

### Western blot

Extraction of proteins from tissue samples or cells using RIPA buffer and protein concentration was measured by the BCA method (Tiangen, Beijing, China). 15 μg proteins were separated by SDS–PAGE and transferred to PVDF membranes (Millipore). Membranes were blocked with 5% skim milk and immunoblotted with primary antibodies, followed by incubation with matched secondary antibodies. Then blots were visualized using an enhanced chemiluminescence kit (Amersham, Little Chalfont, UK) and detected using the GelCapture version software (DNR Bio-Imaging Systems, Jerusalem, Israel). GAPDH was employed as an endogenous control.

### Apoptosis assay

1 × 10^6^ cells were harvested after transfection for 24 h and resuspended in Annexin V binding buffer. 5 μl FITC-Annexin V and 5 μl PI were added to stain using the Apoptosis Detection Kit (BD Biosciences, San Jose, CA). Then, 400 μl PBS was added to the cells, which were analyzed using a FACScan flow cytometry system (BD Biosciences, San Jose, CA). Cell apoptosis was analyzed using FlowJo V7 software (Tree Star, Ashland, OR).

### Luciferase reporter assay

Luciferase reporter assay was performed using psiCHECK2 vector (Promega). To construct the psiCHECK2-ETS2 recombinant vector, the complete 3’UTR of human ETS2 mRNA containing the putative or mutative miR-30a-5p binding sites was amplified and cloned into the psiCHECK2 vector. For the lncRNAs, wild or mutative full-length sequences of lncRNA DLEU2 were amplified and cloned into the psiCHECK2 vector. AGS cells were co-transfected with one of the psiCHECK2 recombinant vectors and miR-30-5p mimics, or miR-NC by Lipofectamine 2000 according to the manufacturer’s guidelines. The relative luciferase activity was valued using the Dual-Luciferase Reporter Assay System (Promega) and Infinate M200 PRO microplate reader (Tecan, Shanghai, China).

### Immunohistochemistry staining

Tissue samples were fixed in formalin for 24 h at 4 °C and embedded in paraffin. The paraffin block was cut into 4 μm sections. Tissue sections were deparaffinized in Van-Clear (Hongci., Shanghai, China) and concentration gradient ethanol, then microwaved in 0.01 M citrate buffer for 10 min. After blocking with 5% goat serum for 1 h at room temperature, the sections were incubated with the primary antibody and then incubated with enzyme-labeled goat anti-mouse/rabbit IgG polymer (160101405L, Maixin., Shanghai, China). The immune response was visualized by the enhanced DAB chromogenic kit (1,705,252,031, Maixin., Shanghai, China), and hematoxylin was used for counterstaining. The immunostaining score was evaluated blindly by two independent investigators as the product of positive staining cell ratio (R) and staining intensity score (S). R was divided into four levels: 0 (< 5%, negative), 1 (5–25%, sporadic), 2 (25–50%, focus), 3 (> 51%, diffuse). S was also divided into four levels: 0 (negative), 1 (weak), 2 (middle), 3 (strong). A total of 0–3 was considered to be low expression, while 4–9 was considered to be high expression. Finally, images were collected by a vertical microscope system (Nikon, Japan).

### Statistical analysis

Statistical analyses were performed with SPSS software version 22.0 (IBM Corp., Armonk, NY). The experimental results were expressed as mean ± standard deviation. The differences between groups were calculated using the Student's t-test or one-way ANOVA. The correlation among lncRNAs, miRNAs, and mRNAs was analyzed by Pearson's correlation analysis. A statistically significant threshold was defined as *P* < 0.05.

## Results

### DLEU2 is up-regulated in GC tissues and cell lines

**Fig. 1:**
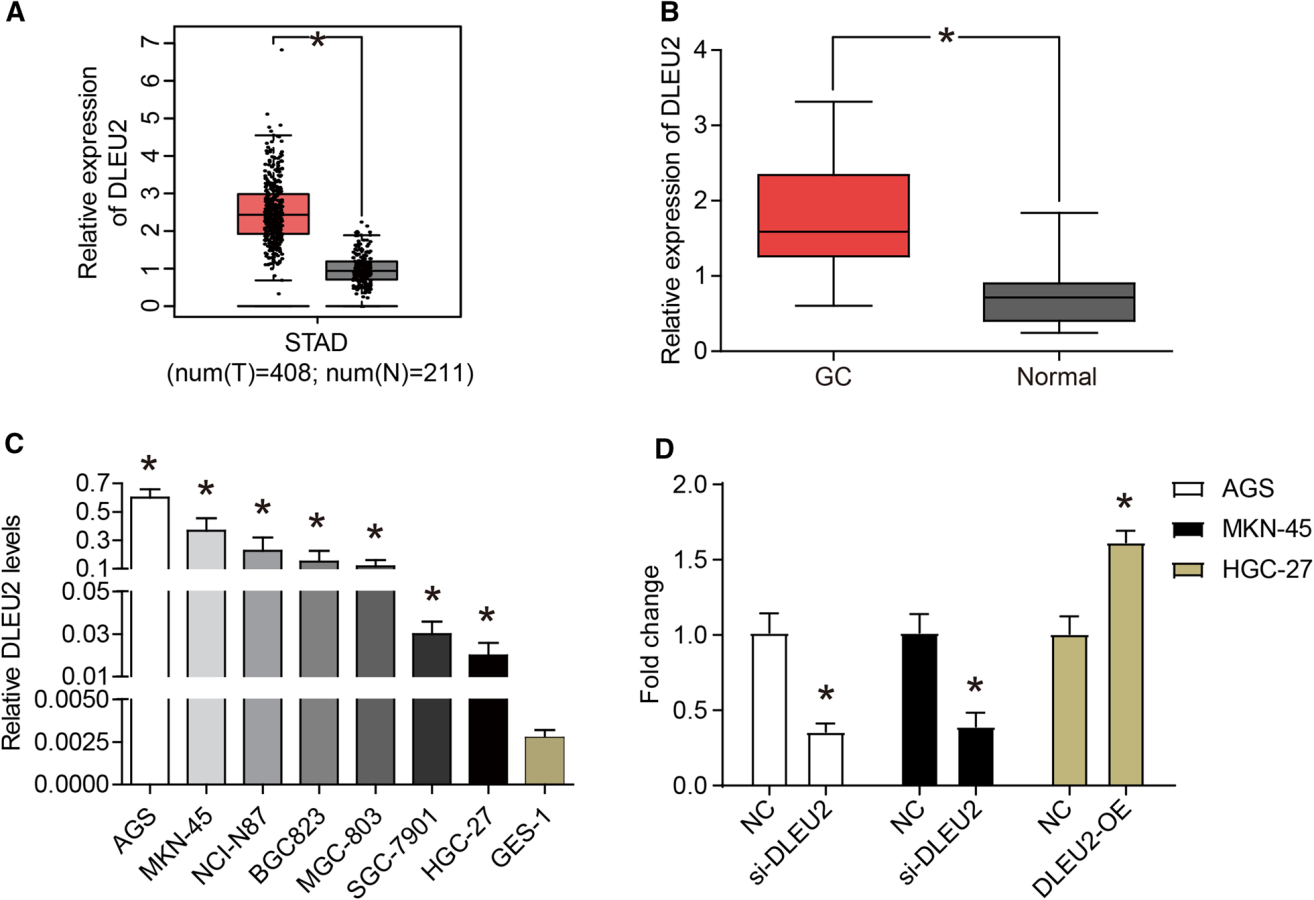
DLEU2 is up-regulated in GC tissues and cell lines. **A** The boxplot of lncRNA DLEU2 level. Red and gray boxes represent stomach adenocarcinoma (STAD) and normal tissues, respectively. The data came from the GEPIA database. **B** LncRNA DLEU2 levels in gastric cancer (GC) tissues and adjacent normal tissues (n = 75) were evaluated via qRT-PCR. **C** LncRNA DLEU2 levels in GC cell lines (MKN-45, SGC-7901, HGC-27, NCI-N87, AGS, BGC823, and MGC-803) and normal gastric mucosal epithelial cell line (GES-1) were evaluated via qRT-PCR. **D** The siRNA targeting DLEU2 (si-DLEU2) was transfected into AGS and MKN-45 cells, and the pcDNA3.1-DLEU2 plasmid (DLEU2-OE) was transfected into HGC-27 cells. After transfection for 24 h, the level of DLEU2 was detected via qRT-PCR. *P < 0.05

GEPIA collected the RNA expression information of 408 stomach adenocarcinoma (STAD) and 211 normal tissues. The analysis results of GEPIA showed that DLEU2 was highly expressed in STAD tissues compared with normal tissues (Fig. 1A). In addition, the expression of DLEU2 in 75 pairs of GC tissues and matched normal tissues was examined using qRT-PCR, and the results showed that expression of DLEU2 was significantly up-regulated in GC tissues (Fig. 1B). Statistical analysis showed that the expression of DLEU2 was significantly associated with the pathological grading (*P* = 0.0087) and TNM stage (*P* = 0.0382) of GC patients (Table 1). No significant correlation with other characteristics was observed, such as age, gender, and tumor diameter. Subsequently, the expression levels of DLEU2 in GC cell lines (MKN-45, SGC-7901, HGC-27, NCI-N87, AGS, BGC823, and MGC-803) and normal gastric mucosal epithelial cell line (GES-1) were detected using qRT-PCR. The results were shown in Fig. 1C, the expression of DLEU2 in all GC cell lines was significantly higher than that in a normal gastric mucosal epithelial cell line. In summary, DLEU2 was significantly up-regulated in GC tissues and cell lines.

**Table 1:** DLEU2 expression associated with the clinicopathological parameters in GC

Clinicopathological parameters	n	DLEU2 expression	P
Gender			
Male	55	1.437 ± 0.0617	0.5541
Female	20	1.500 ± 0.0764	
Age (years)			
< 60	33	1.507 ± 0.0731	0.8911
≥ 60	42	1.493 ± 0.0549	
Tumor diameter (cm)			
< 5	31	1.473 ± 0.0727	0.1748
≥ 5	44	1.623 ± 0.0549	
Pathological grading			
I–II	23	1.406 ± 0.0632	0.0087*
III–IV	52	2.067 ± 0.1004	
TNM staging			
T1–T2	15	1.997 ± 0.1695	0.0382*
T3–T4	60	1.417 ± 0.0869	

*P < 0.05

### DLEU2 promotes the malignant phenotype of GC cells

DLEU2 was expressed at higher levels in AGS and MKN-45 cells compared with other GC cell lines. Therefore, the expression of DLEU2 in AGS and MKN-45 cells was down-regulated using siRNA to explore the roles of DLEU2 in GC cells. After transfection of siRNA targeting DLEU2 (si-DLEU2), the expression of DLEU2 was down-regulated by approximately 60% compared with the negative control (NC) (Fig. 1D). Furthermore, the expression level of DLEU2 in the HGC-27 cell line was lower than other GC cell lines. Therefore, the pcDNA3.1 plasmid was used to over-express DLEU2 in the HGC-27 cells (Fig. 1D). After transfection of overexpression plasmid (DLEU2-OE), the expression of DLEU2 was up-regulated compared with the NC group (Fig. 1D).

**Fig. 2:**
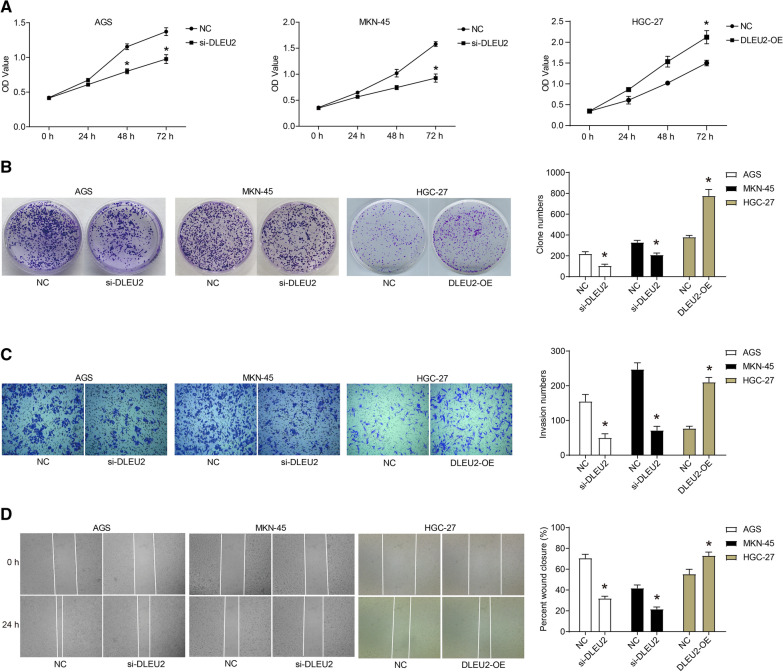
DLEU2 promotes the malignant phenotype of GC cells. The siRNA targeting DLEU2 (si-DLEU2) was transfected into AGS and MKN-45 cells, and the pcDNA3.1-DLEU2 plasmid (DLEU2-OE) was transfected into HGC-27 cells. **A** The proliferation was determined via CCK8 assay. **B** The clonogenicity was detected by colony formation assay. **C** The invasion was determined by transwell assay. **D** The migration was determined by wound healing assay. *P < 0.05

Knockdown of DLEU2 significantly inhibited the proliferation of AGS and MKN-45 cells, as evaluated by CCK-8 assays (Fig. 2A). In parallel, the results of the colony formation assay showed that compared with the NC group, the colony formation ability of AGS and MKN-45 cells transfected with si-DLEU2 was significantly suppressed (Fig. 2B). In addition, the overexpression of DLEU2 significantly promoted the proliferation and clone formation of HGC-27 cells (Fig. 2A, B).

**Fig. 3:**
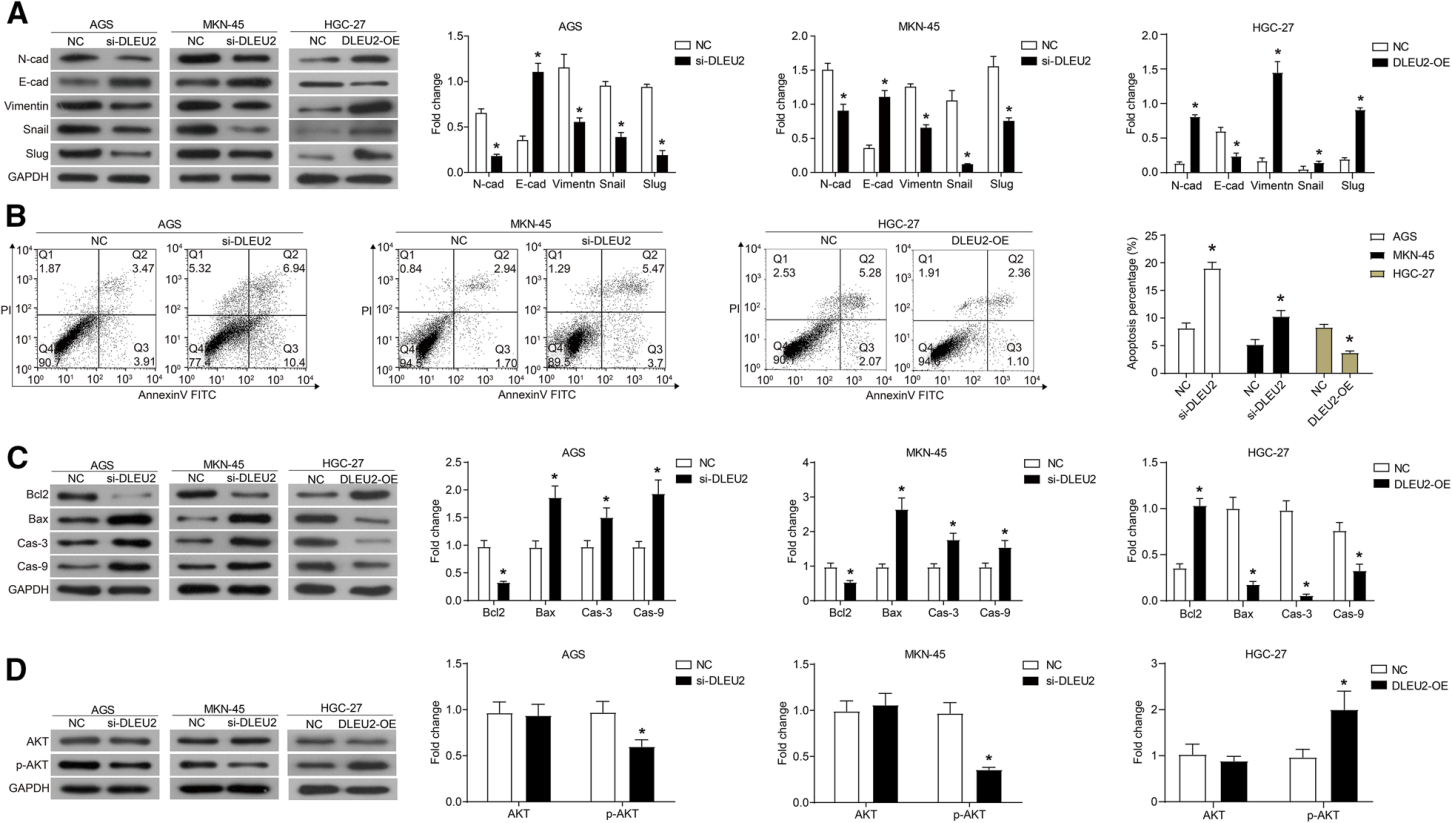
DLEU2 promotes the EMT process and inhibits apoptosis of GC cells. The siRNA targeting DLEU2 (si-DLEU2) was transfected into AGS and MKN-45 cells, and the pcDNA3.1-DLEU2 plasmid (DLEU2-OE) was transfected into HGC-27 cells. **A** The expression of EMT-related proteins was detected by western blot. **B** The apoptosis was detected by flow cytometry. **C** The expression of apoptosis-related proteins was detected by western blot. **D** The activation of the AKT signaling pathway was detected by western blot. *P < 0.05

Moreover, the migration and invasion of GC cells were evaluated using transwell and wound healing assays. We observed that si-DLEU2 transfection inhibited the migration and invasion of AGS and MKN-45 cells, and the overexpression of DLEU2 significantly promoted the migration and invasion of HGC-27 cells (Fig. 2C, D). Furthermore, the expression of E-cadherin was up-regulated, while the protein levels of N-cadherin, Vimentin, Snail, and Slug were down-regulated in AGS and MKN-45 cells transfected with si-DLEU2 (Fig. 3A). In contrast, the expression of E-cadherin was downregulated, while the expression of N-cadherin, Vimentin, Snail, and Slug was up-regulated in HGC-27 cells that overexpressed DLEU2 (Fig. 3A).

Apoptosis analysis revealed that the down-regulation of DLEU2 induced the apoptosis of AGS and MKN-45 cells (Fig. 3B), while the up-regulation of DLEU2 inhibited the apoptosis of HGC-27 cells. Western blot analysis showed that in AGS and MKN-45 cells transfected with si-DLEU2, the expression of Bax increased, caspase 3 and caspase 9 were cleaved, and the expression of Bcl2 decreased (Fig. 3C). In HGC-27 cells that overexpressed DLEU2, the expression of apoptosis-related proteins was contrary to the above description (Fig. 3C).

### DLEU2 acts as a tumor promoter through the AKT signaling pathway

To comprehensively elucidate the mechanisms of DLEU2 in regulating GC cell proliferation, migration, invasion, and apoptosis, we studied the activation of the AKT signaling pathway. We found that after transfection of si-DLEU2, the phosphorylation of AKT was reduced, and the expression of AKT did not change significantly in AGS and MKN-45 cells (Fig. 3D). Moreover, after overexpression of DLEU2, the phosphorylation of AKT increased in HGC-27 cells (Fig. 3D). In summary, DLEU2 upregulated the activation of AKT signaling pathway in GC cells.

### DLEU2 acts as a ceRNA targeting ETS2 via miR-30a-5p

**Fig. 4:**
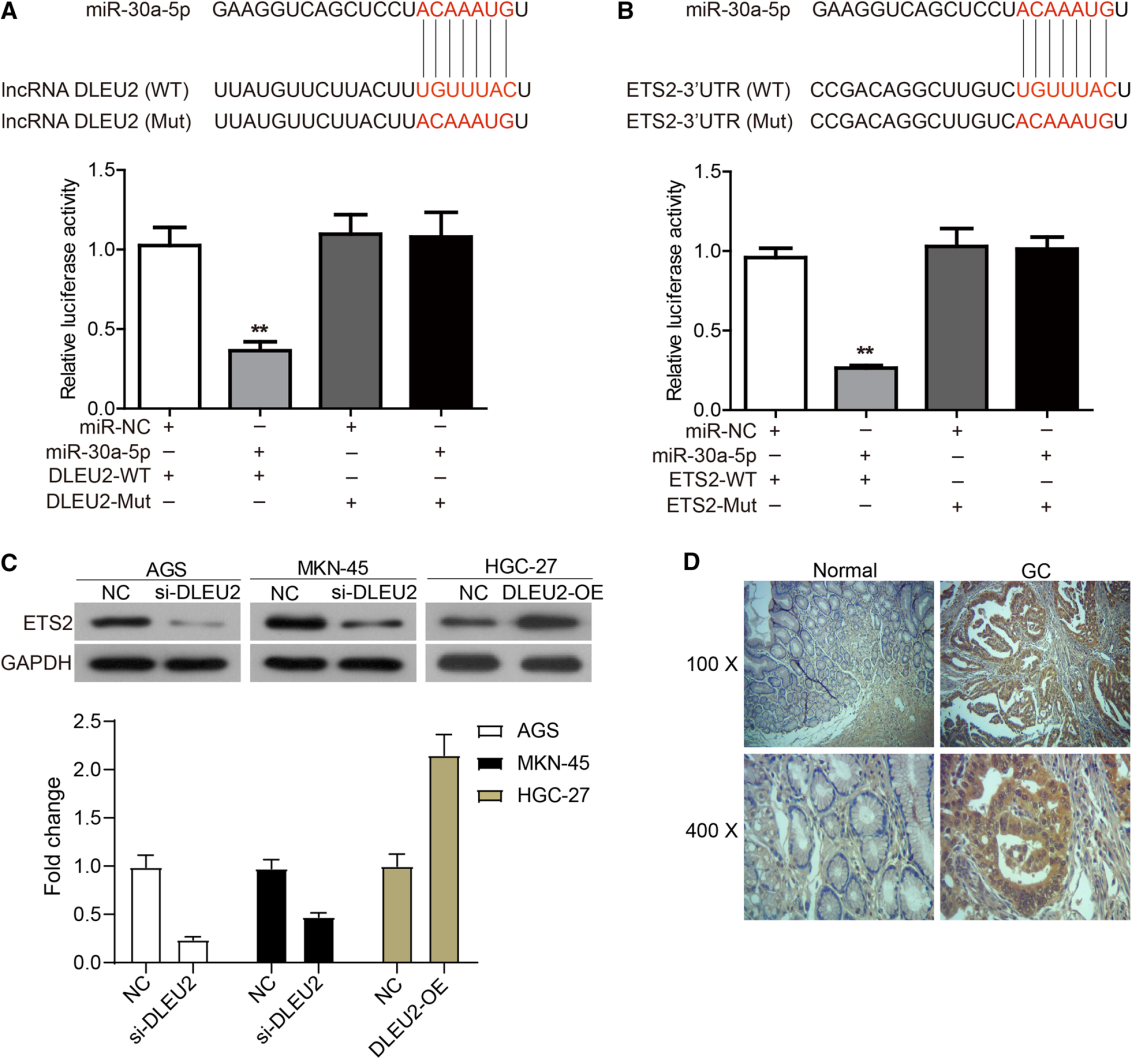
DLEU2 acts as a ceRNA targeting ETS2 via miR-30a-5p. **A** Up: The sequences of miR-30a-5p, wide type of DLEU2 (WT), and mutated lncRNA DLEU2 (Mut). Down: The expression levels of luciferase in AGS cells transfected with wild-type (WT) or mutated (Mut) DLEU2 reporters plus miR-30a-5p mimic or miR-NC were determined. **B** Up: The sequences of miR-30a-5p, wide type of ETS2 (WT), and mutated ETS2 (Mut). Down: The expression levels of luciferase in AGS cells transfected with wild-type (WT) or mutated (Mut) ETS2 reporters plus miR-30a-5p mimic or miR-NC were determined. **C** The expression of ETS2 in AGS and MKN-45 cells transfected with si-DLEU2, and in HGC-27 cells transfected DLEU2 overexpression plasmid (DLEU-OE). **D** The expression of ETS2 in GC tissues and adjacent normal ovarian tissues was evaluated via immunohistochemistry. *P < 0.05

Starbase, an online databse of miRNA-target interactions (http://starbase.sysu.edu.cn/) predicted that DLEU2 could act as a ceRNA to target ETS2 via miR-30a-5p. We verified this in AGS cells (Fig. 4A-D). As shown in Fig. 4A, AGS cells co-transfected with miR-30a-5p mimics and DLEU2-WT showed less luciferase activity than other groups. In parallel, AGS cells co-transfected with miR-30a-5p mimics and ETS2-WT revealed lower luciferase activity than the other groups (Fig. 4B). In addition, the expression of ETS2 was down-regulated in AGS and MKN-45 cells transfected with si-DLEU2, and its expression was up-regulated in HGC-27 cells that overexpressed DLEU2 (Fig. 4C).

We then verified the expression of ETS2 in GC tissues. As shown in Fig. 4D and Table 2, ETS2 was highly expressed in GC tissues (58/75, 77.3%), compared to normal tissues (15/75, 20%, *P* < 0.001). In addition, the association between ETS2 expression and clinical characteristics of patients was analyzed. As shown in Table 3, the expression of ETS2 was significantly correlated with age (*P* = 0.041), pathological grading (*P* < 0.001) and TNM stage (*P* = 0.023). No significant correlation with other characteristics was observed, such as gender and tumor diameter.

**Table 2:** ETS2 expression in GC compared with para-carcinoma tissue

Group	n	ETS2 expression		P
		Low (n%)	High (n%)	
GC	75	17 (22.7)	58 (77.3)	0.001^**^
Para-carcinoma	75	60 (80.0)	15 (20.0)	

*P < 0.05

**Table 3:** ETS2 expression associated with the clinicopathological parameters in GC

Clinicopathological parameters	n	ETS2 Low (n%)	ETS2 High (n%)	P
Gender				
Male	55	11 (20.0)	44 (80.0)	0.800
Female	20	6 (30.0)	14 (70.0)	
Age (years)				
< 60	33	11 (33.3)	22 (66.7)	0.041^*^
≥ 60	42	5 (11.9)	37 (88.1)	
Tumor diameter (cm)				
< 5	31	9 (29.0)	22 (71.0)	0.409
≥ 5	44	8 (18.2)	36 (81.8)	
Pathological grading				
I–II	23	12 (52.2)	11 (47.8)	0.001^**^
III–IV	52	5 (9.6)	47 (90.4)	
TNM staging				
T1–T2	15	7 (46.7)	8 (53.3)	0.023^*^
T3–T4	60	10 (16.7)	50 (83.3)	

*P < 0.05

### DLEU2 exerts its role by regulating the miR-30a-5p/ETS2 axis

**Fig. 5:**
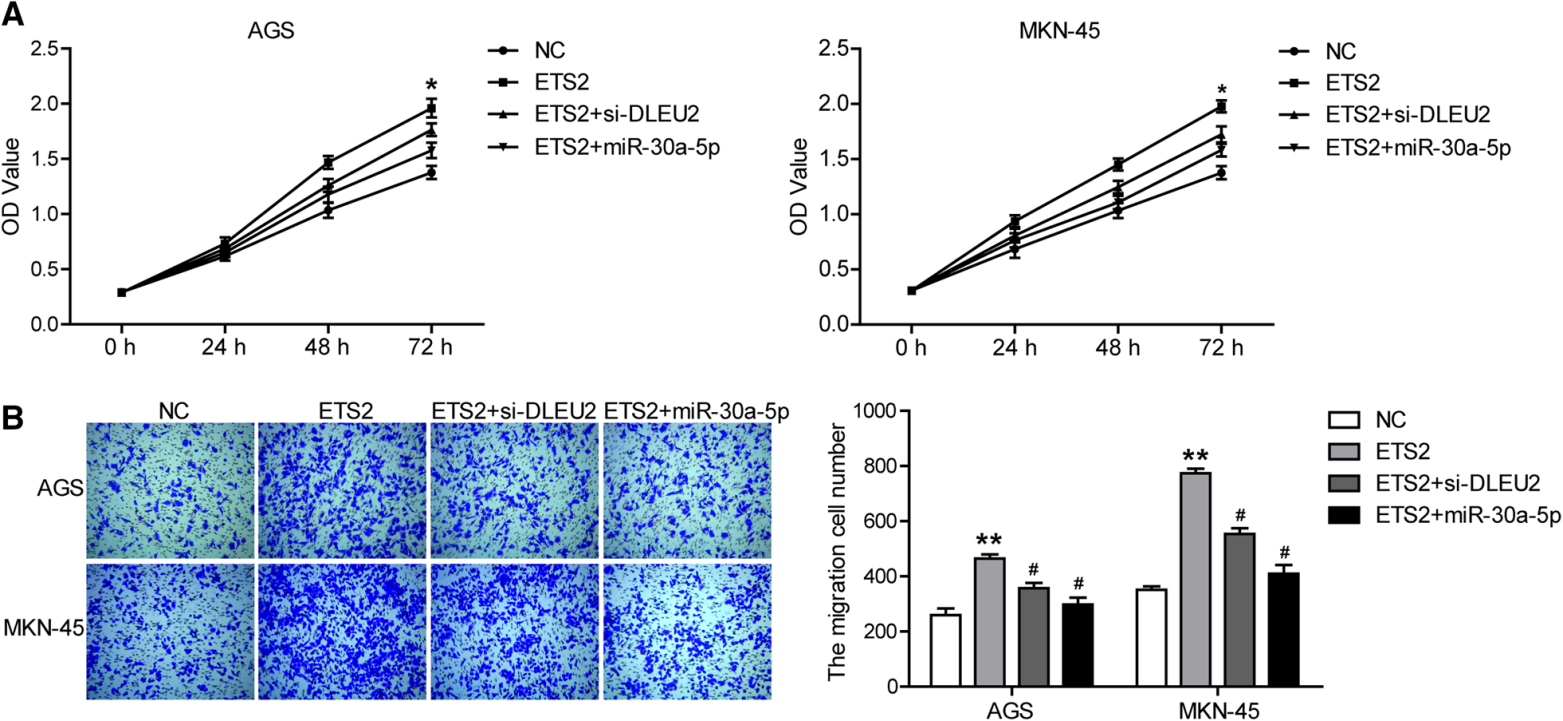
DLEU2 exerts its role by regulating the miR-30a-5p/ETS2 axis. AGS and MKN-45 cells were transfected with pcDNA3.1-ETS2 overexpression plasmid (ETS2), pcDNA3.1-ETS2 plasmid and miR-30a-5p mimics (ETS2 + miR-30a-5p), pcDNA3.1-ETS2 plasmid and si-DLEU2 (ETS2 + si-DLEU2), respectively. **A** The proliferation was determined via CCK8 assay. **B** The invasion was determined by transwell assay. *P < 0.05

Finally, to test whether DLEU2 exerted its role by regulating the miR-30a-5p/ETS2 axis, cells were transfected with pcDNA3.1-ETS2 overexpression plasmid (ETS2), pcDNA3.1-ETS2 plasmid and miR-30a-5p mimics (ETS2 + miR-30a-5p), pcDNA3.1-ETS2 plasmid, and si-DLEU2 (ETS2 + si-DLEU2), respectively. The results showed that the overexpression of ETS2 promoted the proliferation (Fig. 5A) and invasion (Fig. 5B) of AGS and MKN-45 cells. Furthermore, ETS2 overexpression rescued the inhibition of proliferation (Fig. 5A) and invasion (Fig. 5B) induced by DLEU2 down-regulation and miR-30a-5p up-regulation in AGS and MKN-45 cells. These results indicated that DLEU2 may regulate the process of GC cells through the miR-30a-5p/ETS2 axis.

## Discussion

This study revealed for the first time the expression pattern of DLEU2 in GC and its regulation of GC cell processes. We found that DLEU2 was significantly up-regulated in GC tissues and cell lines. Its high expression was significantly associated with the pathological grading and TNM stage of GC patients. This result is consistent with previous research reports that also used RT-qPCR to detect the expression pattern of DLEU2 in other types of tumor tissues and cell lines.DLEU2 also is found to be highly expressed in other types of tumors, such as non-small cell lung cancer [23], glioma [24], esophageal cancer [25, 26], osteosarcoma [27], and hepatocellular carcinoma [28, 29] tissues and cell lines.

Furthermore, we used siRNA and pcDNA3.1 plasmid transfection to knock down and exogenously express DLEU2 in GC cells respectively, and found that knockdown of DLEU2 inhibited the proliferation of AGS and MKN-45 cells, and induced cell apoptosis. Overexpression of DLEU2 promoted the proliferation of HGC-27 cells and inhibited cell apoptosis. Other studies using the same method to knock down and exogenously express DLEU2 in other types of tumor cells resulted in similar conclusions that DLEU2 promotes the malignant proliferation of tumor cells [23, 24].

The epithelial-mesenchymal transition (EMT) is a critical step in tumor progression, which increases cell infiltration and promotes the occurrence of distant metastases [30]. It is characterized by the loss of epithelial markers and the acquisition of mesenchymal markers [31]. In this study, we found that down-regulation of DLEU2 inhibited the migration and invasion of AGS and MKN-45 cells, while the up-regulation of DLEU2 promoted the migration and invasion of HGC-27 cells. Furthermore, increased E-cadherin expression, and decreased N-cadherin, Vimentin, Snail, and Slug expression were observed in si-DLEU2 transfected cells, and decreased E-cadherin expression, and increased N-cadherin, Vimentin, Snail, and Slug expression were observed in DLEU2-OE transfected cells, which suggests that DLEU2 can promote the aggressiveness and motility of tumor cells by regulating the EMT of GC cells. Zhou et al. also used western blot to detect the effect of DLEU2 expression changes on the expression level of EMT markers and find that DLEU2 accelerated the EMT of non-small cell lung cancer cells [23].

In the present study, we found that miR-30a-5p could directly bind to the 3’ UTR region of ETS2, thereby inhibiting the translation and protein stability of ETS2. Moreover, DLEU2 bound to miR-30a-5p through the same binding site, which facilitated the expression of ETS2. Knockdown of DLEU2 reduced the protein level of intracellular ETS2. The *ETS2* gene is located on chromosome 21q22.1-q22.3 with a span of 17.6 kb, which has no TATA box or CAAT box in its promoter and has a major CpG island at its 5’ untranslated region [32]. The ETS2 protein consists of 469 amino acids and has an N-terminal pointed domain and a C-terminal DNA-binding domain. It also has a MAPK phosphorylation site at Thr72, which may mediate transcriptional regulation [33]. As a transcription factor, ETS2 is responsible for the transcriptional regulation of a variety of tumor-associated genes, such as Cyclin D1 [22]. In addition, mutations in the core promoter of the telomerase reverse transcriptase (TERT) gene create a de novo binding site for ETS2, providing a mechanism for cancer-specific telomerase reactivation [34, 35]. In most cancer cells, telomerase reactivation is a ubiquitous process and one of the main features of carcinogenesis [36]. In human cancers, TERT promoter mutations have been shown to define a subpopulation of patients with a poor prognosis [34, 35]. Moreover, the preferential binding of ETS2 to gain-of-function mutant p53 (mut-p53) improves the tumor-promoting role of mut-p53[37]. The above results indicated that ETS2 is involved in the malignant progression of tumor cells through a variety of mechanisms. Furthermore, DLEU2 exerts a cancer-promoting function by regulating the expression of ETS2.

In addition to affecting ETS2, DLEU2 is used as ceRNA to regulate the expression of SOX9, PDK3, E2F7, and other target proteins [23, 24]. ETS2 is a representative member of the ETS family and plays crucial roles in cell proliferation, differentiation, development, and transformation. In the present study, we found that ETS2 was highly expressed in GC, which was consistent with the expression of ETS2 in esophageal squamous cell cancer [38]. The down-regulation of ETS2 significantly reduces the level of p-AKT in renal cell carcinoma cells [16]. Consistent with our results, knockdown of DLEU2 inhibited the AKT phosphorylation. We speculate that DLEU2 may affect the phosphorylation of AKT by regulating the expression of ETS2. Further studies are needed to elucidate this mechanism.

## Conclusions

In conclusion, our data revealed that DLEU2 was highly expressed in GC tissues and its expression was statistically correlcated with the clinicopathological characteristics of GC patients. DLEU2 promoted the proliferation, migration, and invasion of GC cell as well as induced the EMT process and inhibited apoptosis. We found that DLEU2 acted as a ceRNA targeting ETS2 via miR-30a-5p. Furthermore, the AKT signaling pathway was required. Taken together, we suggested that DLEU2 is a potential molecular target for GC treatment.

## Data Availability

The data supporting the conclusions of this paper are included within the manuscript.

## References

[1] Bray F, Ferlay J, Soerjomataram I, Siegel RL, Torre LA, Jemal A (2018). Global cancer statistics 2018: GLOBOCAN estimates of incidence and mortality worldwide for 36 cancers in 185 countries. CA Cancer J Clin.

[2] Miller KD, Nogueira L, Mariotto AB, Rowland JH, Yabroff KR, Alfano CM, Jemal A, Kramer JL, Siegel RL (2019). Cancer treatment and survivorship statistics, 2019. CA Cancer J Clin.

[3] Smyth EC, Nilsson M, Grabsch HI, van Grieken NC, Lordick F (2020). Gastric cancer. Lancet.

[4] Djebali S, Davis CA, Merkel A, Dobin A, Lassmann T, Mortazavi A, Tanzer A, Lagarde J, Lin W, Schlesinger F (2012). Landscape of transcription in human cells. Nature.

[5] Furuno M, Pang KC, Ninomiya N, Fukuda S, Frith MC, Bult C, Kai C, Kawai J, Carninci P, Hayashizaki Y (2006). Clusters of internally primed transcripts reveal novel long noncoding RNAs. PLoS Genet.

[6] Zong W, Ju S, Jing R, Cui M (2018). Long non-coding RNA-mediated regulation of signaling pathways in gastric cancer. Clin Chem Lab Med.

[7] Whitehead J, Pandey GK, Kanduri C (2009). Regulation of the mammalian epigenome by long noncoding RNAs. Biochim Biophys Acta.

[8] Sun TT, He J, Liang Q, Ren LL, Yan TT, Yu TC, Tang JY, Bao YJ, Hu Y, Lin Y (2016). LncRNA GClnc1 promotes gastric carcinogenesis and may act as a modular scaffold of WDR5 and KAT2A complexes to specify the histone modification pattern. Cancer Discov.

[9] Shao Y, Ye M, Jiang X, Sun W, Ding X, Liu Z, Ye G, Zhang X, Xiao B, Guo J (2014). Gastric juice long noncoding RNA used as a tumor marker for screening gastric cancer. Cancer.

[10] Gupta RA, Shah N, Wang KC, Kim J, Horlings HM, Wong DJ, Tsai MC, Hung T, Argani P, Rinn JL (2010). Long non-coding RNA HOTAIR reprograms chromatin state to promote cancer metastasis. Nature.

[11] Tay Y, Rinn J, Pandolfi PP (2014). The multilayered complexity of ceRNA crosstalk and competition. Nature.

[12] Peng W, Si S, Zhang Q, Li C, Zhao F, Wang F, Yu J, Ma R (2015). Long non-coding RNA MEG3 functions as a competing endogenous RNA to regulate gastric cancer progression. J Exp Clin Cancer Res.

[13] Fan ZY, Liu W, Yan C, Zhu ZL, Xu W, Li JF, Su L, Li C, Zhu ZG, Liu B (2016). Identification of a five-lncRNA signature for the diagnosis and prognosis of gastric cancer. Tumour Biol.

[14] Garding A, Bhattacharya N, Claus R, Ruppel M, Tschuch C, Filarsky K, Idler I, Zucknick M, Caudron-Herger M, Oakes C (2013). Epigenetic upregulation of lncRNAs at 13q14.3 in leukemia is linked to the In Cis downregulation of a gene cluster that targets NF-kB. PLoS Genet.

[15] Migliazza A, Bosch F, Komatsu H, Cayanis E, Martinotti S, Toniato E, Guccione E, Qu X, Chien M, Murty VV (2001). Nucleotide sequence, transcription map, and mutation analysis of the 13q14 chromosomal region deleted in B-cell chronic lymphocytic leukemia. Blood.

[16] Wu DM, Wen X, Han XR, Wang S, Wang YJ, Shen M, Fan SH, Zhang ZF, Shan Q, Li MQ (2018). Role of circular RNA DLEU2 in human acute myeloid leukemia. Mol Cell Biol.

[17] Liu Y, Zhu J, Ma X, Han S, Xiao D, Jia Y, Wang Y (2019). ceRNA network construction and comparison of gastric cancer with or without Helicobacter pylori infection. J Cell Physiol.

[18] Du X, Liu B, Luan X, Cui Q, Li L (2018). miR-30 decreases multidrug resistance in human gastric cancer cells by modulating cell autophagy. Exp Ther Med.

[19] Liu X, Ji Q, Zhang C, Liu X, Liu Y, Liu N, Sui H, Zhou L, Wang S, Li Q (2017). miR-30a acts as a tumor suppressor by double-targeting COX-2 and BCL9 in *H. pylori* gastric cancer models. Sci Rep.

[20] Wei GH, Badis G, Berger MF, Kivioja T, Palin K, Enge M, Bonke M, Jolma A, Varjosalo M, Gehrke AR (2010). Genome-wide analysis of ETS-family DNA-binding in vitro and in vivo. EMBO J.

[21] Buggy Y, Maguire TM, McDermott E, Hill AD, O'Higgins N, Duffy MJ (2006). Ets2 transcription factor in normal and neoplastic human breast tissue. Eur J Cancer.

[22] Carbone GM, Napoli S, Valentini A, Cavalli F, Watson DK, Catapano CV (2004). Triplex DNA-mediated downregulation of Ets2 expression results in growth inhibition and apoptosis in human prostate cancer cells. Nucleic Acids Res.

[23] Zhou Y, Shi H, Du Y, Zhao G, Wang X, Li Q, Liu J, Ye L, Shen Z, Guo Y (2019). lncRNA DLEU2 modulates cell proliferation and invasion of non-small cell lung cancer by regulating miR-30c-5p/SOX9 axis. Aging (Albany NY).

[24] Xie Z, Li X, Chen H, Zeng A, Shi Y, Tang Y (2019). The lncRNA-DLEU2/miR-186-5p/PDK3 axis promotes the progress of glioma cells. Am J Transl Res.

[25] Lu T, Wang R, Cai H, Cui Y (2020). Long non-coding RNA DLEU2 promotes the progression of esophageal cancer through miR-30e-5p/E2F7 axis. Biomed Pharmacother.

[26] Ma W, Zhang CQ, Dang CX, Cai HY, Li HL, Miao GY, Wang JK, Zhang LJ (2019). Upregulated long-non-coding RNA DLEU2 exon 9 expression was an independent indicator of unfavorable overall survival in patients with esophageal adenocarcinoma. Biomed Pharmacother.

[27] Liu W, Liu PC, Ma K, Wang YY, Chi QB, Yan M (2020). LncRNA DLEU2 promotes tumor growth by sponging miR-337–3p in human osteosarcoma. Cell Biochem Funct.

[28] Guo Y, Bai M, Lin L, Huang J, An Y, Liang L, Liu Y, Huang W (2019). LncRNA DLEU2 aggravates the progression of hepatocellular carcinoma through binding to EZH2. Biomed Pharmacother.

[29] Salerno D, Chiodo L, Alfano V, Floriot O, Cottone G, Paturel A, Pallocca M, Plissonnier ML, Jeddari S, Belloni L (2020). Hepatitis B protein HBx binds the DLEU2 lncRNA to sustain cccDNA and host cancer-related gene transcription. Gut.

[30] Yu M, Bardia A, Wittner BS, Stott SL, Smas ME, Ting DT, Isakoff SJ, Ciciliano JC, Wells MN, Shah AM (2013). Circulating breast tumor cells exhibit dynamic changes in epithelial and mesenchymal composition. Science.

[31] Yoshida T, Ozawa Y, Kimura T, Sato Y, Kuznetsov G, Xu S, Uesugi M, Agoulnik S, Taylor N, Funahashi Y (2014). Eribulin mesilate suppresses experimental metastasis of breast cancer cells by reversing phenotype from epithelial-mesenchymal transition (EMT) to mesenchymal-epithelial transition (MET) states. Br J Cancer.

[32] Owczarek CM, Portbury KJ, Hardy MP, O'Leary DA, Kudoh J, Shibuya K, Shimizu N, Kola I, Hertzog PJ (2004). Detailed mapping of the ERG-ETS2 interval of human chromosome 21 and comparison with the region of conserved synteny on mouse chromosome 16. Gene.

[33] Basuyaux JP, Ferreira E, Stéhelin D, Butticè GJ (1997). The Ets transcription factors interact with each other and with the c-Fos/c-Jun Complex via distinct protein domains in a DNA-dependent and -independent manner. J Biol Chem.

[34] Heidenreich B, Kumar R (2017). TERT promoter mutations in telomere biology. Mutat Res.

[35] Heidenreich B, Nagore E, Rachakonda PS, Garcia-Casado Z, Requena C, Traves V, Becker J, Soufir N, Hemminki K, Kumar R (2014). Telomerase reverse transcriptase promoter mutations in primary cutaneous melanoma. Nat Commun.

[36] Blackburn EH, Epel ES, Lin J (2015). Human telomere biology: a contributory and interactive factor in aging, disease risks, and protection. Science.

[37] Martinez LA (2016). Mutant p53 and ETS2, a tale of reciprocity. Front Oncol.

[38] Fontana RJ (2003). Management of patients with decompensated HBV cirrhosis. Semin Liver Dis.

